# On the reproducibility of electron-beam lithographic fabrication of photonic nanostructures

**DOI:** 10.1038/s41598-024-58842-w

**Published:** 2024-04-15

**Authors:** Pankaj K. Sahoo, Eve Coates, Callum D. Silver, Kezheng Li, Thomas F. Krauss

**Affiliations:** 1 Photonics Research Group, School of Physics, Engineering and Technology, University of York, York, YO10 5DD UK; 2 Department of Physics, Dhenkanal Autonomous College, Dhenkanal, Odisha 759001 India

**Keywords:** Sensors, Lithography

## Abstract

Photonic nanostructures such as gratings and ring resonators have become ubiquitous building blocks in Photonics. For example, they are used in filters, they resonantly enhance signals and act as grating couplers. Much research effort is invested in using such structures to create novel functionalities, which often employs electron-beam lithography. An intrinsic issue in this field is the ability to accurately achieve a specific operating wavelength, especially for resonant systems, because nanometer-scale variations in feature size may easily detune the device. Here, we examine some of the key fabrication steps and show how to improve the reproducibility of fabricating wavelength scale photonic nanostructures. We use guided mode resonance grating sensors as our exemplar and find that the exposure condition and the development process significantly affect the consistency of the resonance wavelength, amplitude, and sensitivity of the sensor. By having careful control over these factors, we can achieve consistent performance for all the sensors studied, with less than 10% variation in their resonance behaviors. These investigations provide useful guidelines for fabricating nanostructures more reliably and to achieve a higher success rate in exploratory experiments.

## Introduction

Nanostructures add functionality to many areas of Photonics. For example, integrated photonic circuits employ ring resonator filters^1^ for wavelength selectivity, which require tight control over nanoscale gaps; grating couplers^2^ consist of wavelength-scale gratings that need to be carefully controlled for optimum performance. Numerous applications, such as sensing or imaging with resonant metasurfaces, or the entire field of imaging with metalenses^3^ demand a high level of precision in nanofabrication. In the realm of nanofabrication of such devices, it is important to understand the key parameters that determine the fidelity of a given fabrication campaign; even a marginal deviation from the intended geometry can induce notable shifts in the characteristics, especially the resonant wavelength, but also reductions in the quality (Q) factor of resonant devices, or modifications from the target properties, such as the focal length or focusing efficiency of a metalens. While there is an increasing trend to use foundry services with their high level of process control for the fabrication of nanostructured devices, much of the prototyping and research effort still uses electron-beam lithography (EBL). EBL is a complex process with many interacting parameters; to achieve a desired wavelength response and performance, researchers often make multiple devices with slightly varying parameters, or they use post-fabrication tuning or trimming techniques, all of which increase the overhead of device fabrication. Consequently, the accurate determination and control of the nanostructure geometry assumes pivotal significance, which motivated us to perform the detailed study presented here.

As an exemplar for a functional photonic nanostructure, we use wavelength-scale gratings that support guided mode resonances (GMRs). GMR structures exhibit Fano resonances^4–6^ and are typically realized in the near IR wavelength range (λ ≈ 600–800 nm) and exhibit a linewidth of Δλ ≈ 1–3 nm, corresponding to a Q-factor of a few hundred^7^. This Q-factor is sufficiently high for the resonance wavelength to sensitively respond to minute changes in fabrication, yet it is low enough that the resonances can be accurately controlled. For ease of characterization and allowing a large number of samples to be tested, we used the “chirped GMR” approach^8^ whereby the resonance appears as a bright line when imaging the grating onto a CMOS camera. This approach allows us to assess the resonance wavelength, the resonance amplitude and the sensitivity to external refractive index changes, the latter being particularly important for sensing applications. We tested more than 200 gratings and show how consistent performance can be achieved.

## Results

**Figure 1:**
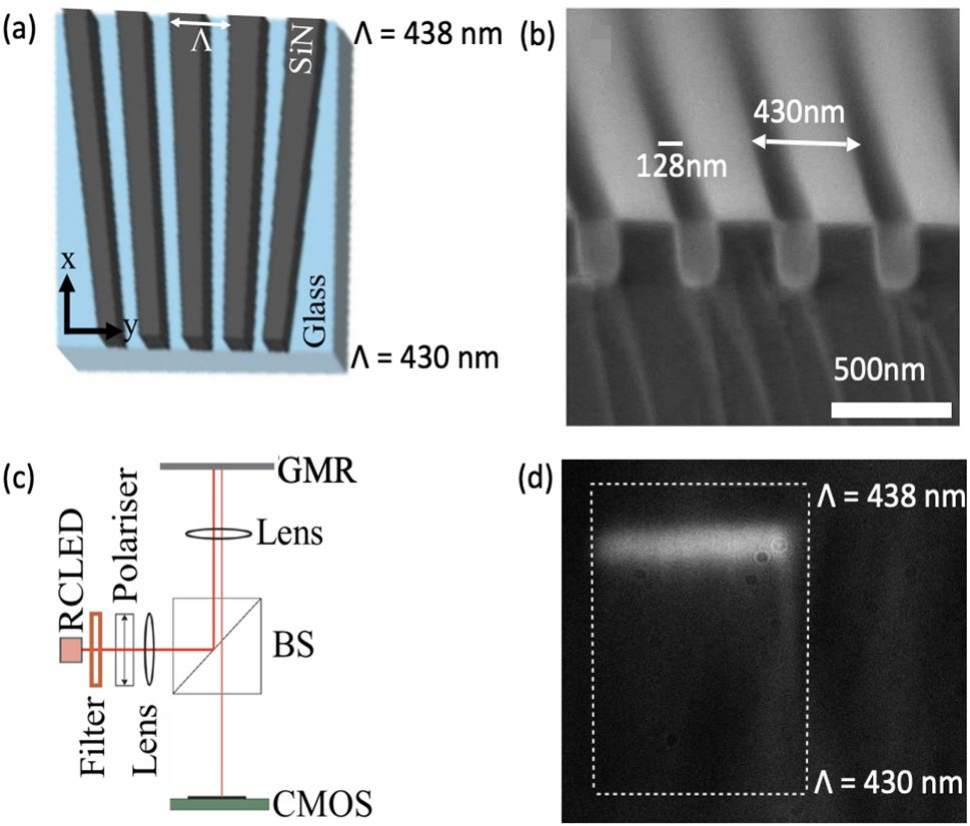
(**a**) Schematic of the Chirped GMR structure; (**b**) SEM cross-section of the grating, with a period of 430 nm and a groove size of 128 nm, so the fill-factor is 0.7; (**c**) Schematic of the set-up used for testing; (**d**) Surface image of the sensor showing the resonance bar at a fixed wavelength of 647 nm.

The chirped GMR sensors^9^ are designed to operate in aqueous media and to resonate around a wavelength of 640-650 nm. A typical structure is shown schematically in Fig. 1a; it is 500 μm × 350 μm in size and it contains gratings of period *Ʌ* that varies between 430 to 438 nm with a fixed fill-factor of 0.7; the fill-factor describes the fraction of high index material. The gratings were fabricated in a 150 nm thick silicon nitride layer on borosilicate glass substrates, so a high fill-factor means that most of the silicon nitride is still there, and the etched grooves are small. An SEM micrograph of a grating with 150 nm etch depth and a fill-factor of 0.7 is shown in Fig. 1b. The resonances are analyzed in a simple imaging setup (schematic in Fig. 1c)^10^, using a filtered RCLED as the light source. The resonance image appears as a bright bar as shown in Fig. 1d.

**Figure 2:**
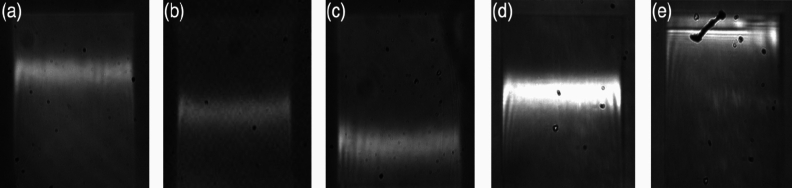
Resonance inconsistency shows the differences in peak position, linewidth, and amplitude as a function of fabrication variations. The field of view is identical to that shown in Fig. 1.

Inconsistencies in the fabrication of these gratings lead to variations in their resonance peak position, peak amplitude (or intensity), linewidth (bar-width) and sensitivity. We show the typical variations we observe from within our large dataset for nominally identical gratings in Fig. 2. Please note that these gratings were made with identical design and that the full range in the vertical position corresponds to a variation of Δλ = 11 nm (from λ = 636 to λ = 647 nm) in nominal resonance wavelength. The fact that the resonance positions vary indicates a fabrication-related change in the grating fill-factor as discussed further below. We also show a simulation of the grating resonance as a function of period and fill-factor in the supplementary section IV to further illustrate this point. Apart from the variation in resonance position, please also note the variation in linewidth and brightness. Here, we study the relevant fabrication parameters and their impact on these variations. We use resonance position as the figure of merit to guide the study and then show that the optimized conditions also reduce the variation in linewidth, resonance amplitude and sensitivity.

The fabrication of resonant gratings is a multistep process which involves (1) Coating of EBL resist, (2) Electron beam exposure, (3) Resist development, and (4) Reactive Ion Etching (RIE). Every step in this sequence contributes to the consistency of the results. The resonance position, our figure of merit, is determined by the period and the fill-factor of the grating. Since the period is accurately determined by the ebeam pattern generator, the fill-factor remains as the main variable, which is affected by all the above four steps. The fill-factor impacts on the optical response of the sensor via the effective index, i.e. a higher fill-factor grating has a higher effective index *n*_*eff*_ than a lower fill-factor grating. The effective index then relates to the resonance position *x* via the following equation. A more detailed explanation of the dependence of the resonance position on the grating parameters is provided in the SI.

1 $$a\left(x\right)=\frac{{\lambda }_{0}}{{n}_{eff}}$$

The function $a\left(x\right)$ is determined by design, and since ${\lambda }_{0}$ is fixed, a change of ${n}_{eff}$ leads to a change in the local resonance period $a\left(x\right)$, which is what we measure and use as our proxy for fabrication consistency. Thus, our primary aim is to track the variation in fill-factor. Let us consider the parameters that determine this fill-factor in more detail; some of them are easily controlled, while others are less so. For example, in step (1), the coating process aims to maintain a constant resist thickness of around 380 nm, which we can achieve consistently by choosing a spin speed of 5000 rpm for 60 s and a baking temperature of 180 °C for 5 min. In step (2), the development time can be fixed to 2 min, using Xylene as the developer, but the development speed (agitation) is more difficult to control. In step (3), the beam current can also be variable, and exposure also depends on the quality of focusing. In step (4), the etch rate is kept fixed by controlling gas flow and pressure, but it also depends on the number of samples (known as the “loading factor”).

To assess the impact of these parameters, one could use SEM or AFM measurements; these are, however, time-consuming, and not necessarily accurate; in particular, SEM measurements depend on the contrast settings used for imaging, while AFM measurements depend on the specific tips used. Instead, we used the optical performance of the gratings, which provides a much more sensitive assessment of quality. Furthermore, optimizing the optical response is the end-goal of the study. In the following sections, we discuss the effects of each parameter in detail.

**Figure 3:**
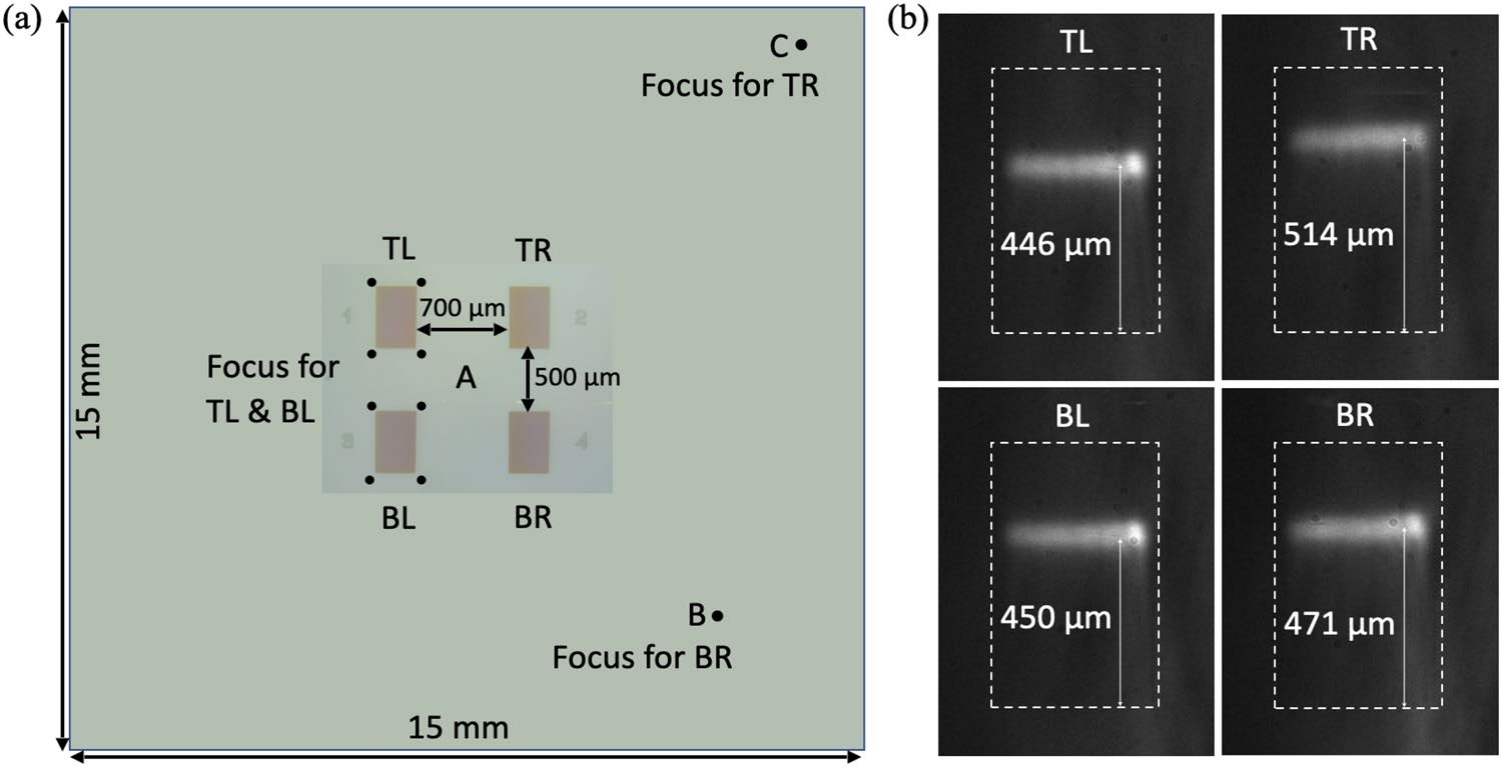
Resist thickness effect (**a**) Four gratings on the same chip for which the beam was focused at different positions. For the two sensors ‘TL and BL’, the beam was focused very close to the respective pattern area (position A). For the sensor TR, the beam was focused near the edge of the wafer which is the farthest point (Point C) from the pattern area. For the sensor BR, the beam is focused midway (Point B) between the pattern and the edge of the wafer, (**b**) The resonance positions for ‘TL and BL’ are the same within error, while the positions for TR and BR have shifted significantly.

The resist thickness has a direct impact on the fill-factor, as it influences the effective exposure dose and the spot size of the focused electron beam. To check this effect, the resist thickness was measured at different locations, namely at the center (A), at an intermediate location (B), and at the edge (C) of the sample (15 mm × 15 mm substrate). The obtained (average) resist thicknesses at A, B, & C are 385 nm, 380 nm, & 360 nm respectively, i.e., the thickness is higher at the center and lower at the edge of the substrate. However, this decrease in thickness is not a linear function of position. The thickness varies slowly in the central part (with a maximum variation of 5 nm within an area of 10 mm × 10 mm) and then drops rapidly towards the edge (with an average change of 20 nm). The complete thickness measurement data are provided in the supplementary section I. To investigate the effect of these thickness variations, four gratings were written on the same chip at four different locations on the substrate namely, the ‘TL’ for ‘Top Left’, ‘BL’ for ‘Bottom Left’ etc. and the beam was focused on different positions. For ‘TL’ and ‘BL’, the beam was focused very close to the respective pattern area indicated by the four dots in Fig. 3a. Note that focusing the beam at four corners makes the focusing process more symmetric to the pattern area. For the sensor ‘TR’, the beam is focused very close to the edge of the wafer (Position C) which is the farthest point from the pattern area. For the sensor ‘BR’, the beam is focused midway between the pattern and the edge of the wafer (Position B). The images of the corresponding resonance positions are given in Fig. 3b. We found that the resonance positions for ‘TL and BL’ are almost the same within a relative shift of less than 5μm, whereas the positions for ‘TR’ and ‘BR’ are shifted by almost 60 and 20 μm, respectively. These observations show that the relative focusing positions and resist thicknesses have a significant impact on the feature size and corresponding resonance position, i.e. far more than we expected at the onset of this study.

**Figure 4:**
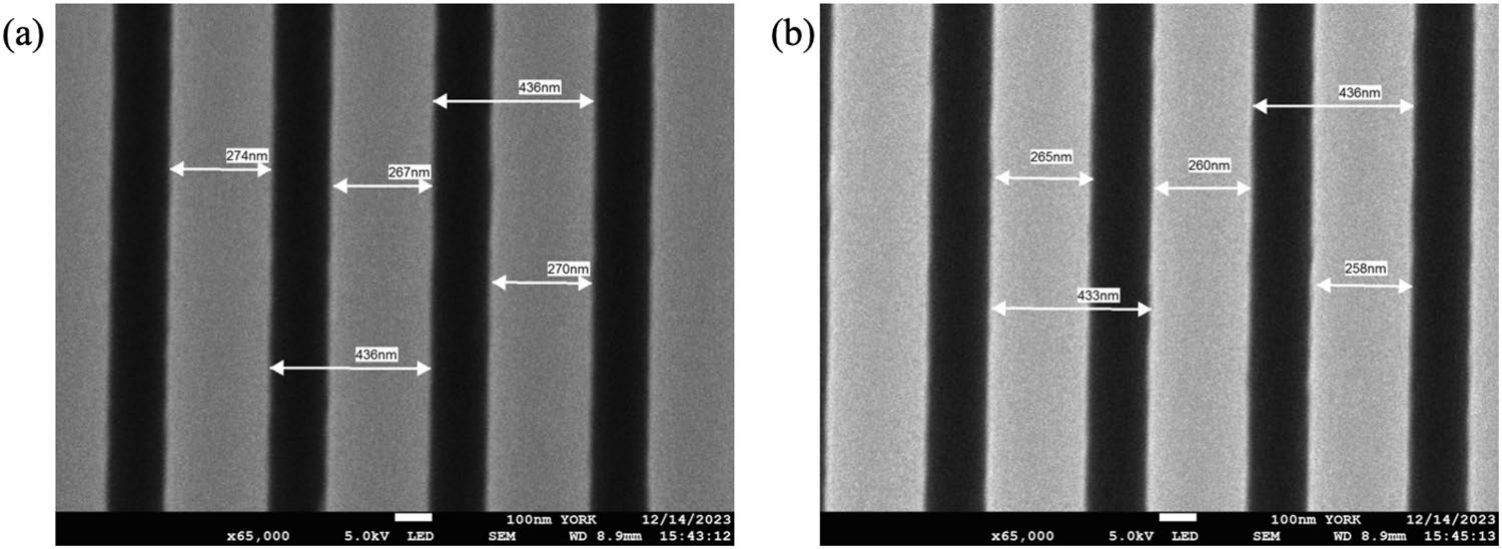
SEM images of grating at (**a**) top left (TL) and (**b**) top right (TR). For an identical period of 436 nm, the grating ridges are approximately 10 nm wider for the TL case, explaining the change in resonance position. Please note that the measurement accuracy is of order of a few nm, as is typical for SEM micrograph analysis.

We can relate these observed differences in resonance position to the fill-factor by conducting careful SEM micrograph studies, choosing the same position on the chirped grating to ensure identity of period. We chose the gratings TL and TR (Fig. 4) because they exhibited the largest difference. We note that for a period of 436 nm, the silicon nitride ridges are approx. 10 nm wider, which explains the observed shift in resonance position by 60µm (see SI, section 2).

**Figure 5:**
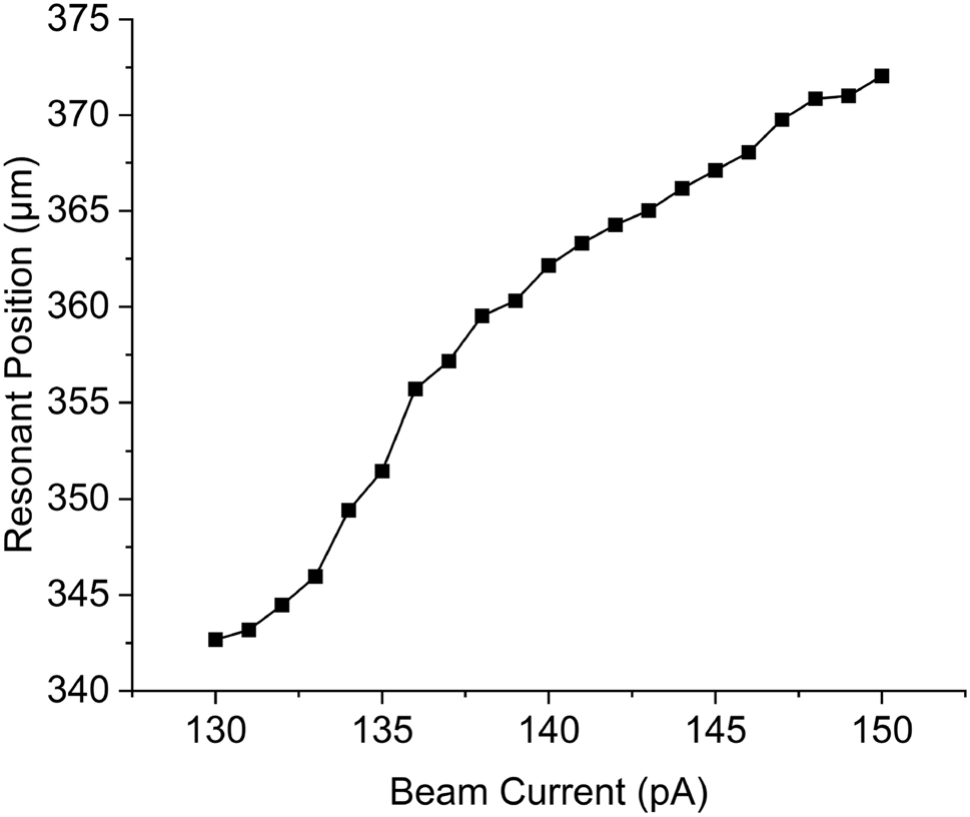
Resonance position Vs Beam current from 130–150 pA.

The electron beam current affects the fill-factor of the grating through the exposure dose, with larger dose resulting in larger grooves, reducing the fill-factor. To investigate this effect, multiple sensors were patterned on the same chip and exposed with beam currents ranging between 130 and 150 pA in steps of 1 pA. The development and etching process are similar for all these sensors as they are exposed on the same chip. As shown in Fig. 5, the resonance position redshifts with a slope of 1.5 μm per pA, which is small, but noticeable.

**Figure 6:**
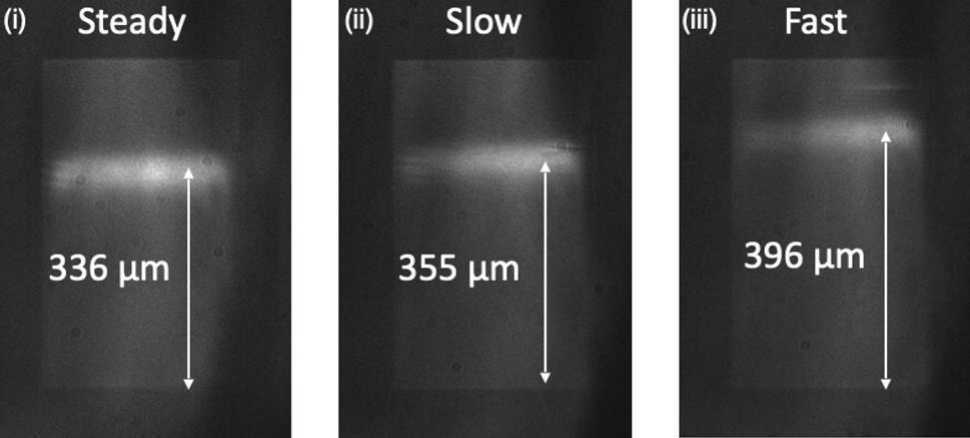
Images of the resonant bar shows the shifting of resonances due to agitation speed.

Exposure and development are interrelated, as short exposure with long or aggressive development can be equivalent to higher exposure dose with short development. The resist development protocol is therefore an important factor that affects the fill-factor of the grating. We use xylene (CAS No. 1330-20-7) as the developer and develop one sample at a time by keeping it horizontal (exposed side facing up) inside a beaker filled with 20 ml of developer. In our process, a development time of 2 min results in the target fill-factor of 0.7, but we note a significant dependence on resist agitation. To investigate the agitation effect, we developed three sensors separately and added a magnetic stirrer into each beaker and setting the speed to three different levels. “Static” refers to 0 rpm, “Slow” to 30 rpm, and “Fast” to 100 rpm. Clearly, increased agitation serves to remove more resist, resulting in larger grooves, so a smaller fill-factor and lower effective index after etching. As before, a lower effective index results in a larger local period *a(x)* according to Eq. 1. We note that (Fig. 6) the resonance position shifts by approximately 20 μm when the speed increases from ‘zero’ to ‘Slow’ (Fig. 6(i),(ii)) and then a further 40 μm when increased to ‘fast’ (Fig. 6 (iii)). In practice, we found that developing without agitation results in a more consistent process. Thus, to maintain resonance consistency, it is essential to keep the liquid motion constant; for simplicity, we decided to opt for the static option.

The dry etching recipe affects the nanostructure through the depth and shape of the grating grooves. Overetching/underetching increases/decreases the air gap, and hence changes the fill-factor of the grating. The gas flow recipe, and etching time significantly affects the etch rate and therefore needs to be controlled carefully to maintain a constant fill-factor. We note that the gas flow and etching time can be indeed controlled accurately; we used a flow of 58 sccm CHF_3_ and 2 sccm O_2_ and a constant time of 7 min, 15 sec to etch the 150 nm of SiN. More importantly, and less obviously, the number of samples in the chamber for a given etch run also affect the etch rate, hence the fill-factor, which is known as the “loading effect”. The loading effect depends on the size of the etching table and the sample, so will vary between different etch tools; our tool has a 7.5 cm diameter table and we use 15 mm square samples. To study the loading effect, we compared the resonance positions of four sensors etched together with that of a single sensor, etched using the same recipe.

**Figure 7:**
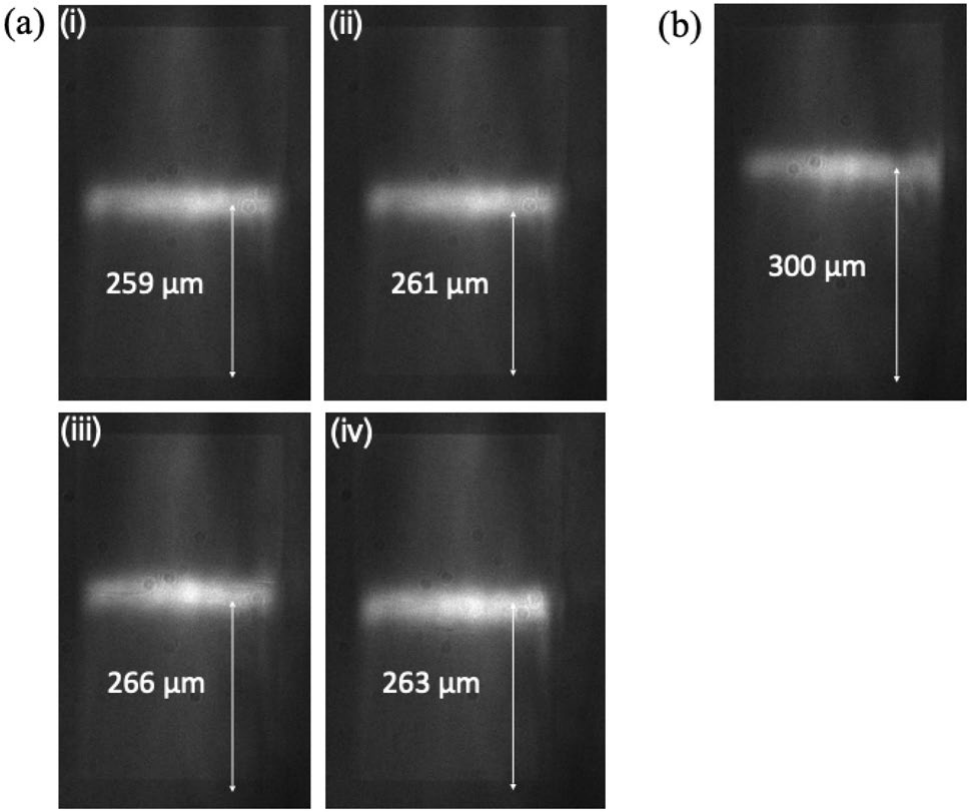
Etching effect (**a**) Four gratings etched together exhibit resonances at similar positions, (**b**) A single grating etched individually has its resonance position significantly farther than the set of four sensors.

The resonance positions of four samples (Fig. 7a (i–iv)) etched together are at very similar position (average = 262 μm), indicating the same fill-factor. In contrast, when a single sample is etched individually for the same duration, the resonance position is shifted by almost 40 μm (Fig. 7b) relative to these four samples. This relative shift indicates that the etch rate for the single sample is faster, hence the grating grooves are slight larger and/or deeper. In consequence, the effective index is lower; according to eq. 1, this means that the local period a(x) where the resonance occurs, for the fixed input wavelength, is larger.

## Statistics of resonance position, amplitude, line width, and sensitivity

**Figure 8:**
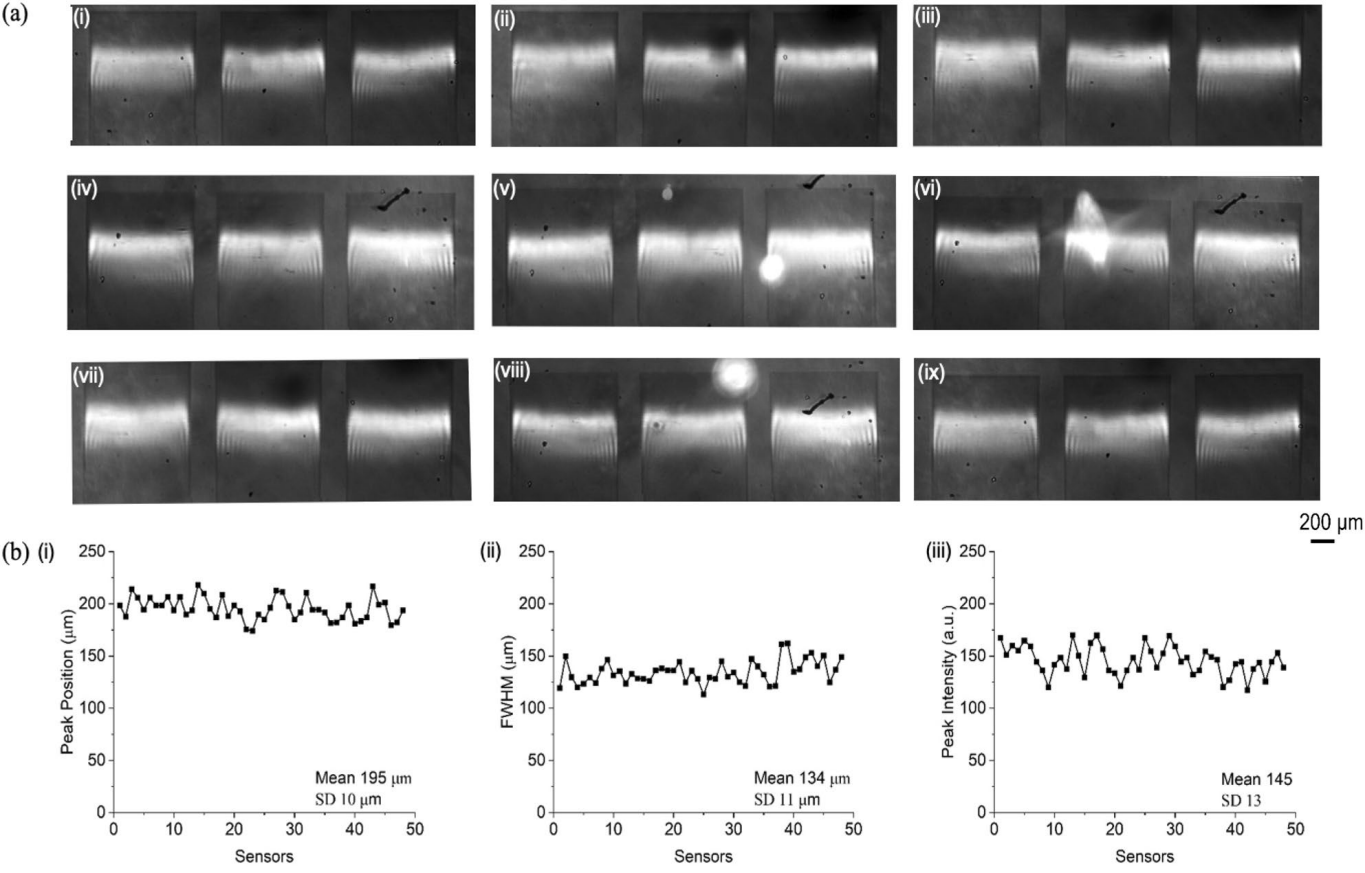
(**a**) Consistency in the resonances of 9 samples fabricated after considering the above effects. (**b**) Statistics showing the mean and SD of Resonance Position, FWHM, Peak Intensity of 50 sensors.

Finally, we tested whether consistency in fill-factor also results in better consistency of all the other relevant parameters of a grating sensor, i.e. Q-factor/linewidth, amplitude, and sensitivity. We show the resonant images of sensors (different batches from single wafer) after homing in on the most promising parameters. We note that the resonance bars are almost at the same position as shown in Fig. 8(a).

A more quantitative analysis on the resonant properties of 50 sensors are obtained and plotted in Fig. 8b. We note that there are only small deviations around the respective mean values of peak position, FWHM (linewidth), and peak intensity (amplitude). The corresponding fill-factor changes only 0.75% around the target fill-factor. Furthermore, the sensitivity performance of 200 such sensors is also evaluated to account for the fact that the figure of merit for photonic sensors depends both on the Q-factor and the amplitude^4^ of the resonance. The obtained sensitivity results are provided in Supplementary section S3.

## Discussion

In conclusion, we have studied the fabrication consistency of photonic nanostructures by using the resonance position of chirped GMR gratings as the model system. We have studied the resonance as a function of the four parameters that we believe impact on the consistency of fabrication most strongly, namely the resist thickness, beam current, development, and etching conditions. From our analysis, we conclude that the consistency of fabrication is significantly affected by the resist thickness and development process. To minimize the inconsistency due to the variation in resist thickness, we suggest focusing the electron beam close to the pattern area, or, better, to ensure that the thickness of the resist film is as uniform as possible. During the post-exposure development process, the agitation of the developer has a similar effect of shifting the resonance position. To maintain the resonance consistency, it is thus important to adopt a consistent development routine that can be easily reproduced; we have chosen to adopt a steady development process where the sample is developed without agitating the solution. Regarding etching, the number of samples etched together also affects the resonance position due to the loading effect, which we observed to be a significant factor. Once all of these parameters are carefully controlled, we note a significant improvement in the resonance consistencies and the consistency improves significantly compared to the low consistency reported^11^ where only 30% of the structures had achieved the target performance. While the detailed parameters will vary between laboratories and fabrication tools, we believe that the guidelines we provide for the consistent fabrication of resonant photonic structures apply more generally, which is imperative for both communications and sensing applications, where the yield of electron beam-based fabrication can be significantly improved.

## Methods

### Materials

SiN-on-glass (Borofloat 33) substrates were bought from Silson Ltd with 150 nm thick Si_3_N_4_. The electron beam resist ARP-13 and charge dissipation layer ARPC-5090 were purchased from ALLResist GmbH (Germany). Microposit^TM^ remover 1165 was obtained from Dupont. Propanol and acetone were purchased from VWR Chemicals. Hydrogen peroxide (35 wt %) and sulphuric acid (95%) were purchased from ThermoFisher Scientific.

### Fabrication of sensors

Clean SiN substrate in Acetone ultrasonic bath for 10 mins, IPA ultrasonic bath for 5 mins, DI water ultrasonic bath for 2 mins. Rinse in DI water, and dry with N_2_. Put the cleaned substrates in oxygen plasma at 200 W, 5 sccm O_2_ for 5 min. The resist ARP13 is spun on SiN at 5000 rpm for 60 s, followed by a bake at 180 °C for 5 mins. The charge dissipation layer ARPC 5090 is spun at 2000 rpm for 60 s, then baked at 90 °C for 2 mins. Electron-beam lithography is performed with a Raith Voyager tool, using an aperture of LC 40 μm, beam current 130 pA, voltage 50 kV to define the GMR with a dose of 145 μC/cm^2^, step size 1 nm in x-direction and 4 nm in y-direction. Remove the ARPC layer following the ebeam exposure by putting in DI for 2 mins. Develop in xylene for 2 mins, followed by IPA for 2 mins, dry with N_2_. RIE etching for 7 minutes with a gas mixture of 12.5 sccm CHF_3_ and 2 sccm O_2_, at 1.8 e^−2^ mtorr, with power 43 W, DC bias 360 V. Put the sample into 1165 resist remover and leave it in an ultrasonic bath for 12 mins, then acetone ultrasonic bath for 3 mins, IPA ultrasonic for 1 min, rinse in DI water and dry with N_2_. Clean the sample in piranha for 5 mins to further remove any residual.

### Characterization of resonant properties

The sensors are illuminated by a RCLED with a 1 nm line filter and a CMOS camera with 2.2 µm pixel size. To achieve the maximum intensity of the resonance on the camera, an integration time of 105 ms was used. The acquisition rate was approximately 1 image every 3 seconds as biological binding happens on a much slower time scale. Given the simplicity and high throughput of the optical path, the spectrally filtered LED provides sufficient signal to produce a high dynamic range image on the CMOS camera.

### Supplementary Information

Supplementary Information.

## Data Availability

The datasets used and/or analyzed during the current study available from the corresponding author on reasonable request.
