# Supplementary material for: On the reproducibility of electron-beam lithographic fabrication of photonic nanostructures
Source: Sci Rep. 2024 Apr 15;14:8703. doi: 10.1038/s41598-024-58842-w (PMC11018749; doi:10.1038/s41598-024-58842-w)
Supplement: Supplementary file 1 — Supplementary Information. [file 41598_2024_58842_MOESM1_ESM.docx]

On the reproducibility of electron-beam lithographic fabrication of photonic nanostructures

# Pankaj K Sahoo^1,2*^, Eve Coates^1^, Callum D Silver^1^, Kezheng Li^1^ and Thomas F Krauss^1^

^1^Photonics Research Group, School of Physics, Engineering and Technology, University of York, UK

^2^Department of Physics, Dhenkanal Autonomous College, Dhenkanal 759001, Odisha, India

*[pankaj.sahoo@york.ac.uk](mailto:pankaj.sahoo@york.ac.uk)

# SUPPLEMENTARY

**I: THICKNESS MEASUREMENT**

To verify the uniformity of the coated resist, the thickness at three positions (refer to Figure 3 (a) in the main manuscript) is measured with a Dektak surface profilometer and shown in Figure S1. The results are described in the main manuscript with relation to the focusing effect.

**
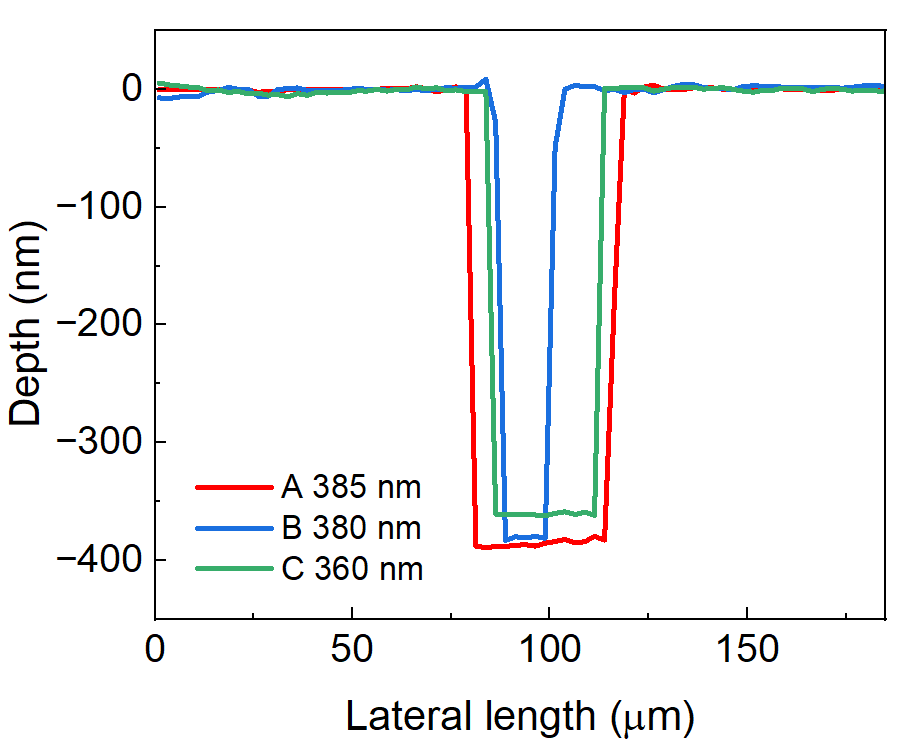
**

Fig. S1
**Fig. S1** Profile data illustrating the resist thickness at various positions, corresponding to Figure 3(a) in the main manuscript. The thickness at Position A is 385 nm, at Position B is 380 nm, and at Position C is 360 nm.

**II: SEM**

Since the chirped GMR gratings were designed with identical fill-factors, any observed variation in fill-factor is fabrication-related. To confirm this assumption, we take SEM images at the centre of each chirped grating (x: 175 μm, y: 250 μm). The fill-factors at four positions: top left (TL), top right (TR), bottom left (BL), and bottom right (BR) were examined and are illustrated in Fig. S2a. Additionally, Fig. S2b displays the resonance position plotted against the fill-factor.

**
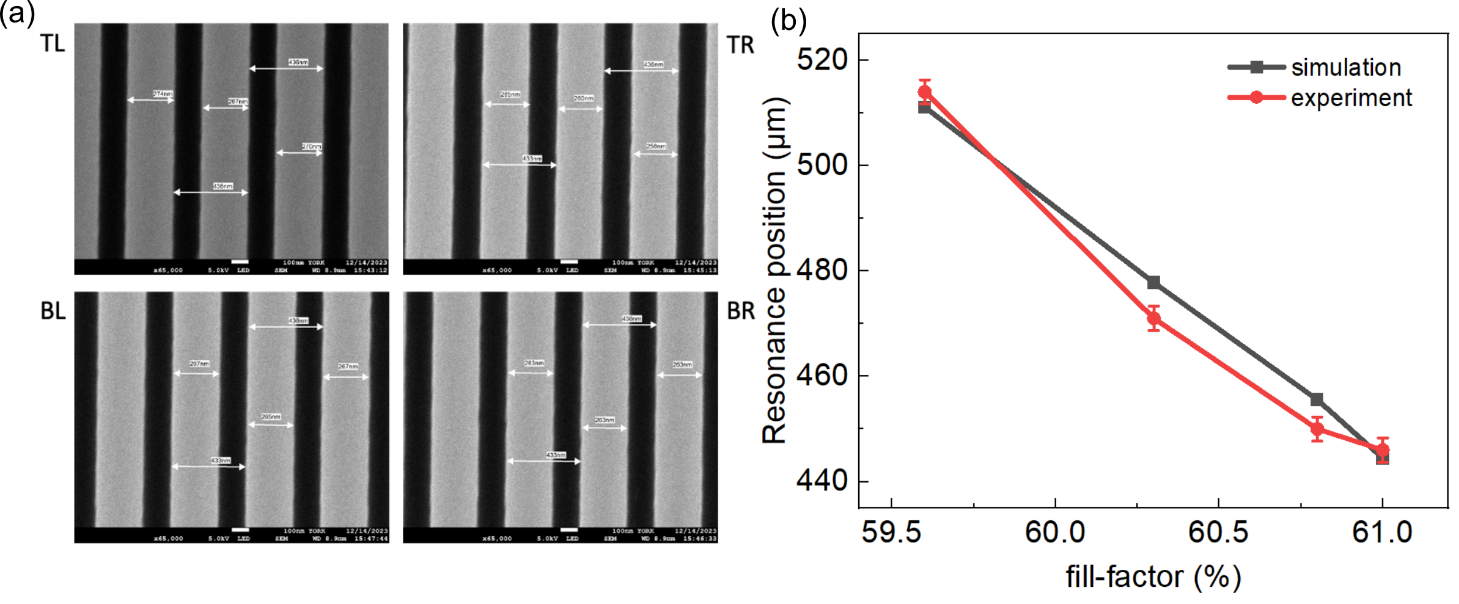
**

**Fig. S2 SEM images and the resonance position vs. fill-factor.** (a) SEM micrographs were captured at four distinct locations with differing resist thicknesses, resulting in varied fill-factors. The period is 436 nm in all cases and the ridge width is 270nm (TL), 260nm (TR), 267nm (BL) and 263nm (BR). The measurement error is estimated to be ± 2.3 nm. (b) The resonance position is depicted as a function of the fill-factor. The simulated results align with the observed trend obtained from measurements.

We find that a shift of around 60 μm in resonance position occurs for an *ff* change of 3%. The measurement data were compared with the simulations using Rigorous Coupled-Wave Analysis (RCWA) and we note good agreement.

To simulate the resonance position, we define the resonance $R$as a function of grating period $P$, refractive index $n$, fill-factor $ff$, material thickness $t$. Here, we used an LED with single output wavelength $\lambda_{0}$. The Resonance of the chirped GMR can be expressed as the function below.

$R=f(P,n,ff,t)=\lambda_{0}$ (1)

The change of the bar position $\Delta x$can be expressed as,

$P_{local}=C_{chirp}\Delta x+P_{initial}$ (2)

Where $P_{initial}$is the initial period of the chirped GMR, and $C_{chirp}$ is the chirping function that describes how fast the period changes, and is defined as

$C_{chirp}=\frac{\Delta P}{L_{chirp}}$ (3)

Where $L_{chirp}$ is the length of the chirped GMR.

First, RCWA simulations were performed to find the period$P$from eq.1 for a given fill-factor $ff$ using the fixed wavelength of $\lambda_{0}=647nm$. By sweeping the fill-factor, we obtain the function of period at 647 nm. Then taking the results obtained from eq.1 into eq.2, we obtain the relationship between position $\Delta x$and period $P$

$\Delta x=\frac{P-P_{initial}}{C_{chirp}}$ (4)

Finally, we plot the position $\Delta x$as a function of fill-factor $ff$.

**III: CONSISTENCY IN SENSITIVITY**

More than 200 sensors were fabricated to analyse the consistency in sensitivity obtained as a result of making the fill-factor consistent. The sensors are designed to operate in aqueous media with three sensor areas of the same parameters (Fig. S3a), allowing for repeat measurements. Each sensor area is 500 μm x 450 μm in size and is separated from the neighbouring sensor by an edge-to-edge gap of 100μm. The sensitivity of the sensors is measured in ethanol dilutions (v/v) with concentrations ranging from 0% (RI 1.3333) to 25% (RI 1.3475). An average resonance shift of 4030 µm per RIU with a standard deviation of 520 µm (12%) is obtained as shown in Fig. S3 (b). This corresponds to a sensitivity of 93 nm/RIU in terms of resonant wavelength.


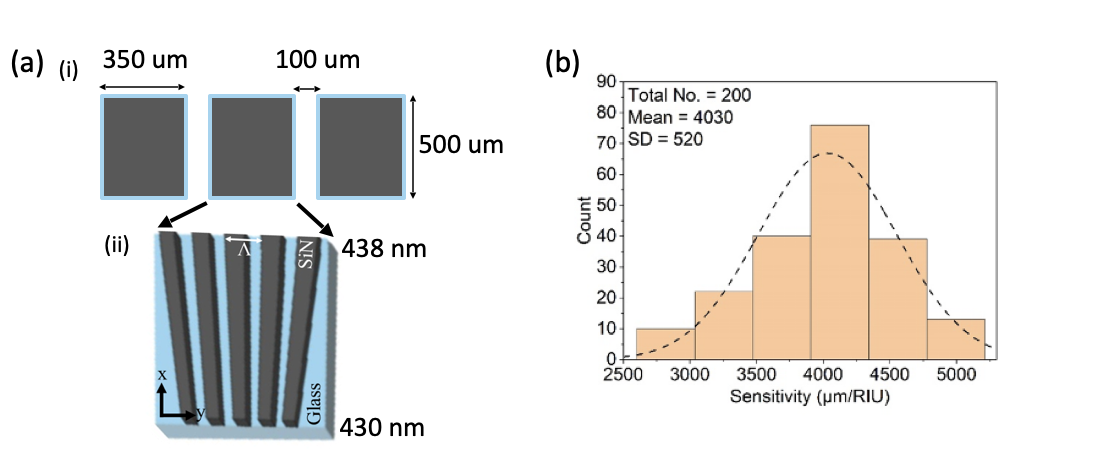


***Fig. S3: (a) Sensor design parameters and the layout, (b) Statistics result showing the mean and standard deviation of the sensitivity for 200 sensors*.**

**IV: SIMULATION OF THE GRATING RESONANCE**


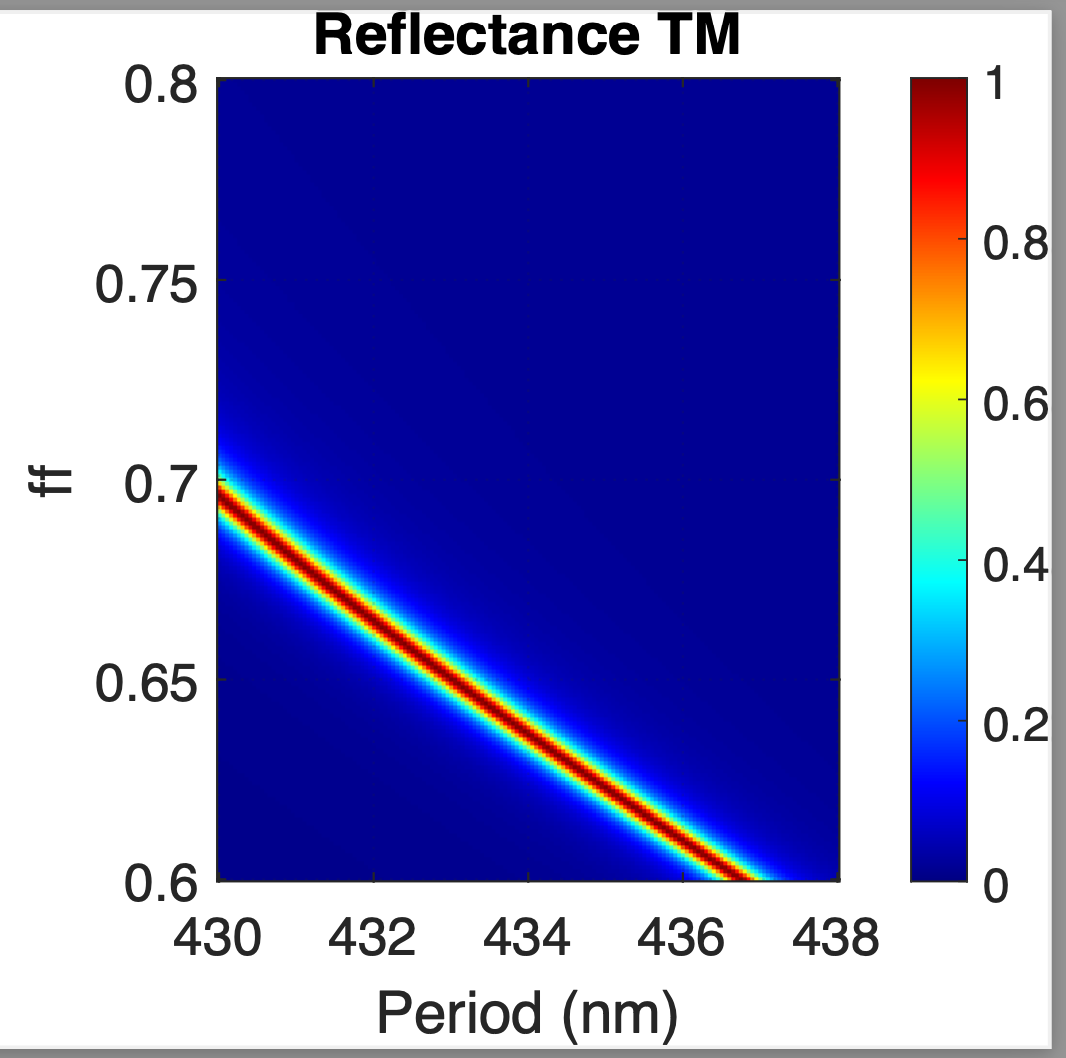


**Fig. S4. Simulation of the grating resonance as a function of grating period and fill-factor. This simulation illustrates how the position of the grating resonance moves to different periods when the fill-factor changes due to fabrication variations.**
